# Nopal feeding reduces adiposity, intestinal inflammation and shifts the cecal microbiota and metabolism in high-fat fed rats

**DOI:** 10.1371/journal.pone.0171672

**Published:** 2017-02-14

**Authors:** Sofia Moran-Ramos, Xuan He, Elizabeth L. Chin, Armando R. Tovar, Nimbe Torres, Carolyn M. Slupsky, Helen E. Raybould

**Affiliations:** 1 Departamento de Fisiologia de la Nutricion, Instituto Nacional de Ciencias Medicas y Nutricion Salvador Zubiran, Mexico D.F; 2 Conacyt, Unidad de Genomica de Poblaciones, Instituto Nacional de Medicina Genomica, Mexico D.F; 3 Department of Food Science and Technology, One Shields Avenue, University of California, Davis, Davis, California, United States of America; 4 Department of Nutrition, One Shields Avenue, University of California, Davis, Davis, California, United States of America; 5 Department of Anatomy, Physiology, and Cell Biology, School of Veterinary Medicine One Shields Avenue, University of California, Davis, Davis, California, United States of America; University of Hawai'i at Manoa College of Tropical Agriculture and Human Resources, UNITED STATES

## Abstract

Nopal is a cactus plant widely consumed in Mexico that has been used in traditional medicine to aid in the treatment of type-2 diabetes. We previously showed that chronic consumption of dehydrated nopal ameliorated hepatic steatosis in obese (*fa/fa*) rats; however, description of the effects on other tissues is sparse. The aim of the present study was to investigate the effects of nopal cladode consumption on intestinal physiology, microbial community structure, adipose tissue, and serum biochemistry in diet-induced obese rats. Rats were fed either a normal fat (NF) diet or a HF diet containing 4% of dietary fiber from either nopal or cellulose for 6 weeks. Consumption of nopal counteracted HF-induced adiposity and adipocyte hypertrophy, and induced profound changes in intestinal physiology. Nopal consumption reduced biomarkers of intestinal inflammation (mRNA expression of IL-6) and oxidative stress (ROS), modfied gut microbiota composition, increasing microbial diversity and cecal fermentation (SCFA), and altered the serum metabolome. Interestingly, metabolomic analysis of dehydrated nopal revealed a high choline content, which appeared to generate high levels of serum betaine, that correlated negatively with hepatic triglyceride (TAG) levels. A parallel decrease in some of the taxa associated with the production of trimethylamine, suggest an increase in choline absorption and bioavailability with transformation to betaine. The latter may partially explain the previously observed effect of nopal on the development of hepatic steatosis. In conclusion, this study provides new evidence on the effects of nopal consumption on normal and HF-diet induced changes in the intestine, the liver and systemic metabolism.

## Introduction

*Opuntia ficus indica*, also known as nopal or prickly pear cactus, is widely consumed in the Mexican diet and used as a traditional medicine in the treatment of metabolic diseases such as diabetes and hypercholesterolemia [1, 2]. Nopal is high in dietary fiber and polyphenols, and is regarded as a functional food due to its low glycemic index and high antioxidant properties [3]. Nopal consumption has been shown to reduce serum triglycerides (TG), C-reactive protein, and glucose intolerance while increasing serum adiponectin concentrations in subjects with metabolic syndrome [4]. We recently demonstrated that chronic consumption of dehydrated nopal ameliorated hepatic steatosis by increasing VLDL secretion in obese (*fa*/*fa*) rats. Furthermore, nopal consumption resulted in lower total serum cholesterol levels and LDL particles. Serum biomarkers of hepatic injury (alanine and aspartate aminotransferase) were significantly lower in the nopal group. These changes suggest an effect of nopal consumption on hepatic function by decreasing TG accumulation and fatty acid peroxidation, thereby reducing hepatic oxidative stress and improving liver insulin signaling [3]. However, the mechanisms underlying the beneficial effects of nopal consumption remain unclear. Whether the effects of nopal are restricted to the liver or could also involve beneficial changes in intestinal and adipocyte function as well as other metabolic markers associated with obesity, is unknown.

The typical western diet, which is high in fat, contributes to the development of metabolic abnormalities in humans; rodents fed high-fat diets (HF diet) are used as models of obesity and metabolic disorders [5]. Studies have demonstrated that chronic ingestion of a HF diet results in increased body weight and adiposity, changes in adipocyte morphology [5], insulin resistance [6], and dyslipidemia. Obesity and long-term consumption of a HF diet are characterized by a low-grade systemic inflammation whose precise etiological origin is unknown [7]. Adipose tissue is one source of inflammation [7] but importantly, recent evidence suggests that a HF diet increases gut permeability [8] and promotes inflammation and oxidative stress in the gastrointestinal tract [9–11]. An increase in intestinal permeability and inflammatory markers may contribute to the presence of circulating pro-inflammatory cytokines [12]. Indeed, there is evidence to suggest that intestinal inflammation precedes the onset of increased body weight or adiposity, suggesting that the gastrointestinal tract plays an etiological role in the response to ingestion of a HF diet [13]. Moreover, intestinal inflammation and an increase in adiposity in response to ingestion of a HF diet are seen in conventionally raised, but not germ-free mice, suggesting that gut microbiota are key players in shaping the phenotypic response to a HF diet [13]. HF diet-induced obesity has also been linked to chronic, low levels of circulating lipopolysaccharide (LPS), a bacterial breakdown product, which may arise from changes in gut microbial composition and increased intestinal permeability [14]. As a consequence, this “metabolic endotoxemia” is thought to drive changes in body weight regulation, glucose tolerance and result in the development of fatty liver [15].

In the present study, we investigated the effects of nopal cladode consumption on HF diet-induced obesity in rats. We hypothesized that the beneficial effects of nopal include amelioration of the harmful effects of a HF diet on the intestine and adipose tissue. Nopal is rich in dietary fiber and polyphenols, and therefore we additionally hypothesized that these functional components shift the composition of gut microbiota, and the cecal and serum metabolomes, which may help to explain the phenotypic improvement seen with nopal consumption. Here, rats were fed either normal fat (NF) or HF diet containing 4% of dietary fiber from either dehydrated nopal cladode or cellulose. Weight gain, food intake, adiposity level, intestinal inflammation and oxidative stress markers, serum biochemical markers and metabolic profiles were measured in response to nopal feeding for 6 weeks. These data were subsequently integrated with the profile of microbial fermentation by-products and microbial composition to provide insight into the molecular mechanism of the beneficial effects of nopal consumption.

## Material and methods

*Nopal*. Fresh nopal cladodes (*Opuntia ficus indica*) were kindly donated by a local grower from Milpa Alta, Mexico (geographic coordinates 19° 13’ 21.6” N, 99° 01’ 32.6” W) and dehydrated at 50°C for 24–36 h. The chemical composition of dehydrated nopal has been previously reported [3].

### Animals and diets

Male Sprague-Dawley rats (initial body wt 235 ± 1 g) were single-housed in a temperature-controlled room with a 12:12-h light-dark cycle and fed either a normal fat diet (NF; Research Diets D12450H, New Brunswick, New Jersey, USA) or a HF diet (Research Diets D12451) for 4 weeks. Diets were administered in pellet form. After 4 weeks, rats on the two diets were then divided into 2 subgroups to consume either NF or NF + nopal (n = 5 for each group) and HF or HF + nopal diet for 6 weeks (n = 14 for each group). The nopal diets were formulated to provide 4% of dietary fiber from nopal in place of cellulose, according to the AIN-93G diet (custom diets, Research Diets). The carbohydrate and protein content of the dehydrated nopal replaced cornstarch and casein in the NF and HF diet. Both NF and NF + nopal diets provided 3.8 kcal/g of energy (72% carbohydrate, 18% protein, 10% fat) whereas HF and HF + nopal provided 4.6 kcal/g of energy (36% carbohydrate, 18% protein, 46% fat). Body weight and food intake were recorded twice a week. Animals were euthanized at 10 weeks. All experiments were performed in accordance with protocols reviewed and approved by the Institutional Animal Care and Use Committee, University of California, Davis.

### Sample collection

At 10 weeks, animals were fasted overnight and deeply anesthetized with isofluorane (Shering, Kenilworth, NJ). Blood was collected by cardiac puncture, centrifuged, and serum was stored at -80°C until further analysis. After blood collection, animals were euthanized by bilateral thoracotomy under deep isoflurane anesthesia. Epididymal, mesenteric, retroperitoneal and subcutaneous fat pads were dissected, weighed, and adiposity index was determined. Samples of fat pads, liver, cecum and colon were rapidly removed, snap-frozen and stored at -80°C until analysis. Additional samples of subcutaneous fat and liver were taken and fixed in formalin for hematoxylin-and-eosin staining. The cecum was removed and its content was weighed and stored at -80°C until analysis.

### Serum biochemical variables

Serum leptin and adiponectin were determined by ELISA, according to manufacturer’s instructions (Alpco Diagnostics, Salem, NH). Serum glucose, triglyceride (TG) and cholesterol were measured using an enzymatic-photometric assay kit (Pointe, Scientific Inc, Canton, MI).

### Histological analysis and adipocyte size quantification

10-μm paraffin embedded sections of subcutaneous fat pads were stained with hematoxylin and eosin. The sections were captured at a total magnification of 100x using the MetaMorph Basic v. 7.7.0. image-analyzer software on an Olympus BX61 microscope (Olympus, Melville, NY). The area of adipocytes was measured with ImageJ 1.42p digital imaging processing software [16]. For the analysis, the images were converted into a binary format, and the area of adipocytes for each sample was analyzed in three random microscopic fields, excluding cells in the borders. The frequency distribution of the adipocyte cell surface area was expressed as percentage of total cells per experimental group.

### Determination of ROS and MPO activity

The formation of ROS in the intestinal tissue was estimated according to Orozco-Ibarra [17]. Briefly, 50 mg of frozen tissue was homogenized in PBS to obtain a final concentration of 100 mg/mL. The homogenized tissue was centrifuged at 6000 g, 4°C for 5 min and the supernatant was collected. 50 μL of a 1:4 dilution of the supernatant was mixed with 50 μL of a 5 μM solution of dichlorofluorescein diacetate and incubated protected from light for 1 h at 37°C. The fluorescence was measured at an excitation wavelength of 488 nm and an emission wavelength of 525 nm (SpectraMax M2, Molecular Devices, Sunnyvale, CA). Fluorescent units were normalized per mg of protein. Myeloperoxidase (MPO) activity was determined as a measure of inflammation and neutrophil infiltration using an *o-dianasidine* assay according to [8]. Briefly, intestinal samples were sonicated over ice for 20 s in 500 μl of 0.5% hexadecyltrimethylammonium bromide in potassium phosphate buffer (pH = 6). Samples were frozen and thawed three times, sonicated for 10 s, and centrifuged (10,000 rpm, 30 min, 4°C), and the pellet was again frozen and thawed. This cycle was repeated two times. The final supernatant (10 μl) was mixed with 290 μl of potassium phosphate buffer containing 0.167 mg/ml of *o-dianasidine* dihydrochloride and 0.005% hydrogen peroxide. Absorbance was read at 450 nm at 2, 3, 4 and 5 min. Activity was expressed as the difference in absorbance units per minute and normalized per mg of protein.

### Determination of inflammation markers using quantitative real-time PCR

Total RNA was prepared using TRIZOL (Invitrogen, Carlsbad, CA) according to the manufacturer’s instructions. Reverse transcription reaction (RT) was performed with 3 mg total RNA from each sample using random primers. PCR assays for each target gene were analyzed in triplicate in a 96-well assay plate with Lightcyler 480 Sequence Detection System (Roche Diagnostics, Indianapolis, IN). PCR primers were obtained from Sigma Aldrich: TNF-α (F:ATG-TGGAACTGGCAGAGGAG, R:GCCATGGAACTGATGAGAGG), IL6 (F:ACCACCCACAACAGACCAGT, R:CGGAA-CTCCAGAAGACCAGA) and MCP-1 (F:CAGTTAATGCCCCACTCACC, R:CCTTATT-GGGGTCAGCACAG). The relative amounts of mRNA were calculated by using the DDCt method with an efficiency adjustment according to the Pfaffl equation [18]. Actin was used as the invariant control.

### Histological analysis and liver lipids

Liver sections, 4 μm thick, were stained with hematoxylin and eosin. A morphologic analysis of liver tissue was performed with sections that were captured at a total magnification of 100x using the MetaMorph Basic v. 7.7.0. image-analyzer software on an Olympus BX61 microscope (Olympus, Melville, NY). Total lipids were extracted using the Folch method [19]. Briefly, 300 mg of frozen tissue were homogenized in 2:1 chloroform:methanol solution. The homogenate was mixed and vortexed with 1.5 mL of 0.9% NaCl and then centrifuged (3,000 rpm, 10 min, 20°C). The organic phase was removed and dried under N_2_. The pellet was resuspended in 10% triton in isopropanol. Triglycerides were quantified using an enzymatic-photometric assay (Diasys Diagnostics Systems GmbH, Holzheim, Germany).

### Nopal and cecal metabolite extraction

Rat cecal samples were weighed in 15 mL tubes, frozen overnight, and lyophilized using a Labconco FreeZone 4.5L Freeze Dry System (Labconco, Kansas City, MO). The average wet weight of cecal samples used for metabolite extraction was 0.404 ± 0.017 g. Dry cecal samples were weighed and stored at -80°C until extraction. The average dry weight of cecal samples was 0.076 ± 0.019 g. Cecal and dehydrated nopal sample metabolites were extracted using a modified version of the Bligh and Dyer method [20].

### Metabolomics analysis

Lyophilized cecal and nopal extracts were diluted in 260–270 μl of phosphate buffer (pH 6.8, 10mM) and centrifuged (10k g, 4°C, 10 min). Serum samples were prepared for NMR analysis as previously described [21]. All metabolomics samples were prepared using 3 mm Bruker NMR tubes with 3-(trimethylsilyl)-1-propanesulfonic acid-d6 (DSS-d6) as an internal standard. Data acquisition was within 24 hours of sample preparation. NMR spectra were acquired as previously described [22] using a Bruker Avance 600 MHz NMR equipped with a SampleJet autosampler at 25°C. NMR spectra were processed and profiled as previously described [22] using Chenomx NMR Suite v7.6 Processor (Chenomx Inc., Edmonton, Canada). Corrections for serum sample dilution were made where appropriate using a dilution factor that was calculated by dividing the final volume of sample by the initial volume of serum used for sample preparation. The concentrations of serum samples are reported in micro-molar concentration (μM). The concentrations of cecal and nopal sample metabolites are reported in mmol/g dry weight.

### Cecal DNA extraction, bacterial 16S rRNA sequencing and data analysis

DNA was extracted from cecal content by a bead-beating method using the ZR Fecal DNA MiniPrep Kit (Zymo Research Corporation) according to the manufacturer’s instructions. The bead-beating lysis step was performed using a FastPrep-24, (MP Biomedicals, Solon, Ohio) homogenizer for 2 min. The V4 region of the 16S rRNA gene was amplified and purified using the procedure described previously [23] and subsequently submitted to the UC Davis Genome Center DNA technologies Core for 250 bp paired-end sequencing on the Illumina Miseq platform. A total of 458,755 paired-end sequences were analyzed using Quantitative Insights Into Microbial Ecology (QIIME) pipeline v.1.7.0. Sequences were filtered to remove low quality sequences with the following parameters: max_bad_run_length = 3, min_per_read_length_fraction = 0.75, phred_quality_threshold = 3, max_barcode_error = 0. Uclust Reference-based Operational taxonomic unit (OTU) selection (closed reference OTU selection method) was used against the most current Greengenes core database (“gg_13_8_otus”). Approximately 31% of sequences failed to be assigned and were therefore discarded. OTUs were defined as bacterial sequences with at least 97% similarity. Taxonomic groups observed in less than 10 counts or fewer than 2 replicate animals were omitted. Due to unequal sequencing depth between different samples, the OTU table was rarified by randomly selecting a subset of 22,270 sequences at the highest sequence count. α-diversity (species richness per group) was assessed by analysis of observed species per sample at the even OTU count of 22,270. The differences in the microbial community structures were explored using an unweighted UniFrac distance followed by Principal Coordinate Analysis (PCoA). Multivariate statistical analysis was performed in the LEfSe package [24] with default parameters.

### Statistical analysis

Values are expressed as mean ± SEM. Except for metabolomics data, data were assessed using the Kolmogorov-Smirnov Z test to examine the distribution type; if data did not exhibit a normal distribution; they were logarithmically transformed prior to analysis. Data were analyzed using GraphPad Prism (version 6.0; Graph Pad Software, Inc. La Jolla, CA). Differences were considered significant when P < 0.05. Two-way ANOVA was used to determine the main effects of nopal on diet (control vs. nopal) and the amount of fat in the diet (NF vs. HF) and their interaction. When the main effects of nopal presence or fat amount were found and there was no significant interaction effect, data were pooled (by nopal or amount of fat in the diet) and Bonferroni post hoc pairwise analysis was performed. When a significant interaction effect was found, differences between all 4 groups were determined using Tukey’s test. For measurements where only HF and HF + nopal groups were evaluated (i.e. for metabolomics measurements), a Wilcoxon-Rank Sum test was performed to evaluate significance. Multivariate statistical analyses for the metabolomics data were performed as previously described [22] using SIMCA-P with mean centering and unit variance scaling. Finally, linear correlations between selected parameters were measured using Pearson’s R coefficient in GraphPad Prism.

## Results

### Metabolomic analysis of nopal

**Fig 1:**
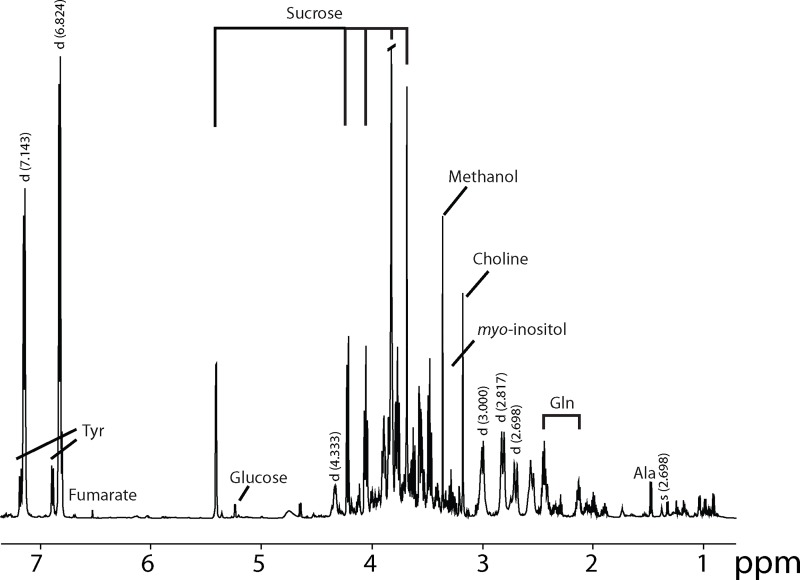
^1^H-NMR spectrum of nopal extract.

To determine the small molecule composition of nopal, ^1^H NMR based metabolomics was used to identify and quantify the water-soluble components (Fig 1). Nopal contains sucrose, choline and, as previously reported [25], amino acids such as glutamine, tyrosine and proline (Table 1). Although methanol was quantified, it was a component of the extraction, and thus may not reflect the true amount present in nopal (Fig 1). Two unidentified peaks with high magnitude (doublets at 7.14, 6.82 ppm) were detected in the aromatic region of the spectrum with similar chemical shifts to the aromatic ring of tyrosine, but were not tyrosine. Given the similarity in chemical shifts in the aromatic region to tyrosine and phenol, these peaks are likely associated with a phenolic type compound that remains unidentified.

**Table 1:** Analysis of dehydrated nopal by ^1^H NMR spectroscopy. Nopal samples were run in duplicate. Data are presented as mean ± SEM.

Compound	Content (μM/g)
4-Aminobutyrate	6.68 ± 0.28
Acetate	0.63 ± 0.04
Alanine	7.38 ± 0.13
Arginine	0.55 ± 0.000
Asparagine	1.92 ± 0.18
Betaine	0.05 ± 0.00
Carnitine	1.28 ± 0.04
Carnosine	1.12 ± 0.04
Choline	9.08 ± 0.39
Ethanol	3.26 ± 0.16
Ethanolamine	2.54 ± 0.10
Formate	0.41 ± 0.00
Fructose	17.58 ± 2.64
Fumarate	1.34 ± 0.01
Galactose	1.46 ± 0.1
Glucose	22.70 ± 0.24
Glutamine	29.52 ± 0.17
Isoleucine	1.01 ± 0.00
Leucine	1.00 ± 0.04
Lysine	0.56 ± 0.16
Malate	0.52 ± 0.09
Methanol	42.83 ± 8.48
Methionine	0.44 ± 0.27
Phenylalanine	1.07 ± 0.03
Proline	22.83 ± 0.83
Putrescine	1.79 ± 0.17
Succinate	0.68 ± 0.06
Sucrose	101.90 ± 1.50
Threonine	2.83 ± 0.08
Tryptophan	2.03 ± 0.06
Tyrosine	23.91 ± 1.18
Valine	3.55 ± 0.08
myo-Inositol	26.35 ± 0.62

### Effect of nopal ingestion on serum biochemical parameters

To investigate the impact of nopal consumption on metabolism, serum TG, cholesterol and glucose concentrations were evaluated (summarized in Table 2). Serum TG concentrations were within normal ranges in all groups [26]; however, they were lower in the HF-fed compared with the NF-fed rats. Serum TG concentration of both groups of nopal-fed rats was significantly higher when compared to rats fed the control NF and HF diets. Cholesterol concentrations were not significantly different with HF diet or nopal consumption, and were within normal range (Table 2). The serum glucose level was not different between the HF and the NF group; however, serum glucose concentration was significantly lower in rats fed with nopal (Table 2). Adiponectin levels did not differ significantly between any of the groups (Table 2).

**Table 2:** Weight gain, energy intake, fasting serum glucose, triglyceride, cholesterol and adiponectin concentration of Sprague-Dawley rats fed NF, NF + nopal, HF or HF + nopal diet for 10 wk.

	Normal-fat diet		High-fat diet		Two-way anova *P* value		
	Control	Nopal	Control	Nopal	Nopal (N)	Fat (F)	NxF
Energy intake (kcal/day)	74.6 ± 1.1	73.0 ± 1.3	74.7 ± 1.8	76.3 ± 1.6	NS	NS	NS
4-wk weight gain before treatment (%)	52.6 ± 2.6	51.3 ± 1.4	52.6 ± 1.6	52.6 ± 1.4	NS	NS	NS
6-wk weight gain after treatment (%)	21.7 ± 1.3	20.3 ± 1.1	20.7 ± 0.6	20.1 ± 0.6	NS	NS	NS
Serum glucose (mg/dL)	155.5 ± 9.2	121.8 ± 6.6	158.5 ± 12.2	138.0 ± 7.5	< 0.05	NS	NS
Serum triglycerides (mg/dL)	63.7 ± 16.5	111.5 ± 19.6	31.0 ± 6.8	47.0 ± 8.1	< 0.05	< 0.0001	NS
Serum cholesterol (mg/dL)	96.8 ± 3.7	84.6 ± 3.4	96.8 ± 3.2	100.7 ± 4.3	NS	NS	NS
Serum adiponectin (μg/mL)	11.9 ± 0.8	11.6 ± 0.8	13.4 ± 0.8	11.7 ± 0.6	NS	NS	NS

Data are presented as mean ± SEM, n = 5–14

*P < 0.05.

### Effect of nopal ingestion on adipocyte and liver physiology

**Fig 2:**
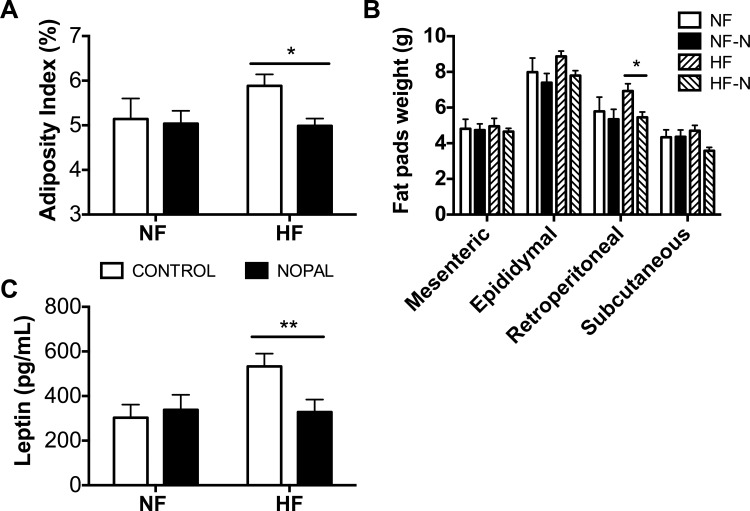
Effect of nopal consumption on adiposity and serum leptin levels. A) Adiposity index. B) Fat pad mass. C) Serum leptin levels. Data are presented as mean ± SEM (n = 5–14 per group). Significance was set at P < 0.05.

Dietary fat and nopal consumption had no effect on overall weight gain (Table 2). However, nopal consumption significantly decreased adiposity in HF-fed rats (Fig 2A). This decrease resulted predominantly from a decrease in retroperitoneal fat pad mass (Fig 2B). In accordance with adiposity, serum leptin concentration was significantly decreased by 38% with nopal ingestion in HF-fed rats (Fig 2C). Leptin plasma levels from all experimental groups were positively correlated with adiposity index (data not shown; p = 0.0009, and R^2^ = 0.3291). There was no difference in daily food intake between the NF and the HF fed rats, or between the nopal fed rats and the controls (two-way ANOVA, Table 2).

**Fig 3:**
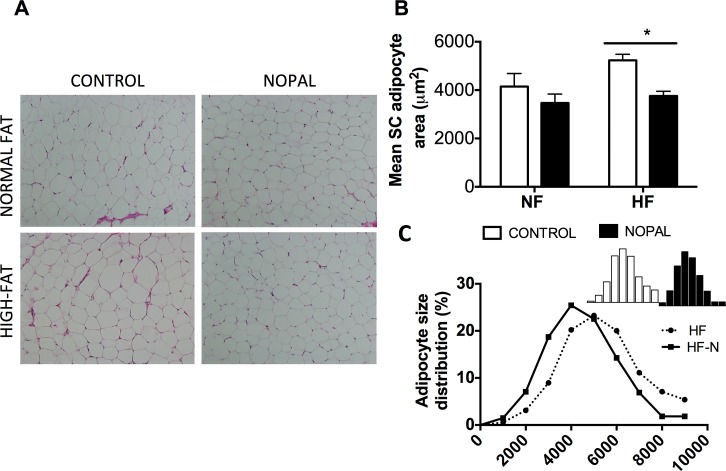
**Effect of nopal consumption on adipocyte morphology** A) Representative photographs with 100x magnification of H&E stained sections of subcutaneous adipose tissue from rats fed NF, NF + nopal, HF, or HF + nopal. B) Mean adipocyte size of subcutaneous adipose tissue. C) Adipocyte size distribution in high fat fed rats. Data are presented as mean ± SEM (n = 5–14 per group). Significance is set at P < 0.05.

Previous reports have shown that greater adipocyte size observed in obesity correlates positively with adipose tissue inflammation [27]. To further investigate the effect of nopal consumption on adiposity, we evaluated the adipocyte size in the subcutaneous fat pads. There was a trend in the size of adipocytes by the degree of dietary fat consumption (P = 0.07). However, adipocyte size was significantly lower in the HF + nopal group compared with HF-fed rats (Fig 3B). The frequency distribution of adipocyte cell surface area also differed between HF and HF + nopal rats. The adipose tissue of rats fed a HF diet had a higher percentage of cells with a higher surface area compared with the rats fed the HF + nopal diet. Approximately 67% of cells had a surface area greater than 4000 μm^2^ in HF fed rats, whereas in the HF + nopal fed group only 48% of cells had a surface area greater than 4000 μm^2^ (Fig 3C).

**Fig 4:**
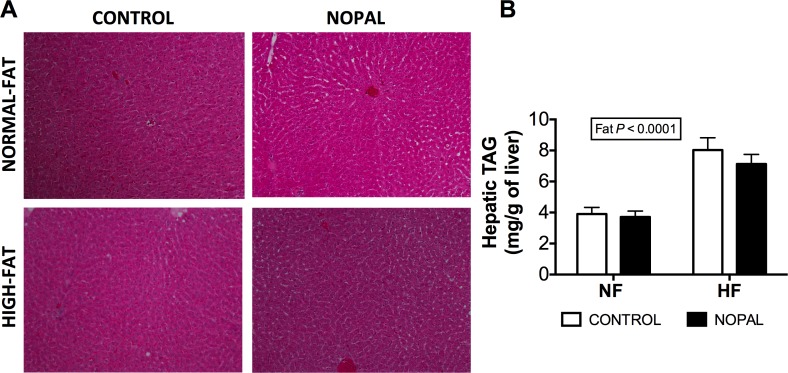
Liver histology of NF, NF + nopal, HF and HF + nopal rats. A) Representative photographs with 100x magnification of H&E stained liver sections of rats fed NF and NF + nopal or HF and HF + nopal diets. B) Hepatic triglycerides levels. Data are presented as mean ± SEM (n = 5–14 per group).

Histological analysis of liver sections using hematoxylin-and-eosin staining showed a normal morphology with no apparent indication of hepatic steatosis in any of the groups (Fig 4A). Rats fed a HF diet had higher concentrations of hepatic TG compared to NF fed rats (Fig 4B) and this observation is in agreement with the previous observations [28]. However, nopal consumption had no significant effect on hepatic TG, although there was a trend toward lower values. The effect of nopal on hepatic TG is not consistent with previous observations using genetically predisposed rat model [3].

### Effect of nopal ingestion on serum metabolome

**Fig 5:**
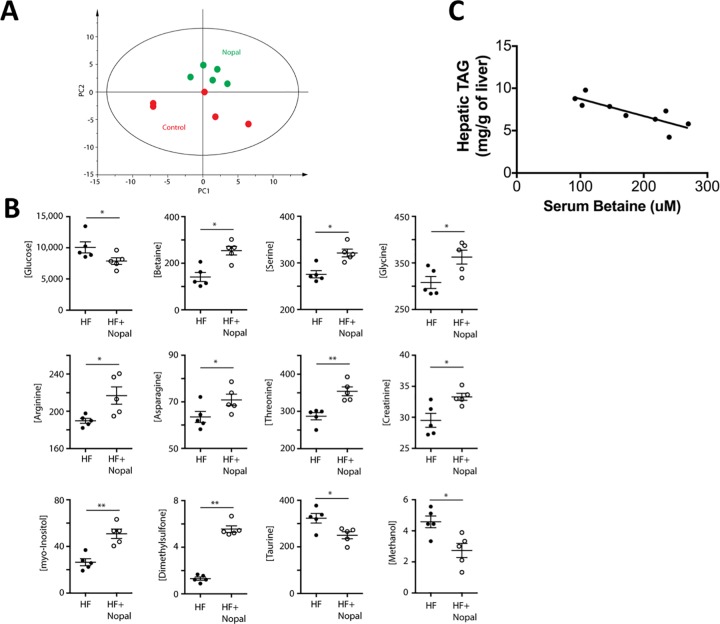
Impact of Nopal on serum metabolites of HF-fed rats. A) Principal Component Analysis (PCA) revealed distinct separation between serum metabolomic profiles from rats fed a HF diet (red) or a HF + nopal diet (green) (R2X = 0.68; Q2 = 0.12). B) Serum metabolite concentrations (in micromoles/L). Significance is based on Mann-Whitney non-parametric analysis, and is set at p < 0.1 (n = 5 per group). C) Serum betaine levels correlate negatively with hepatic triglyceride levels (R^2^ = -0.67; p = 0.007).

**Fig 6:**
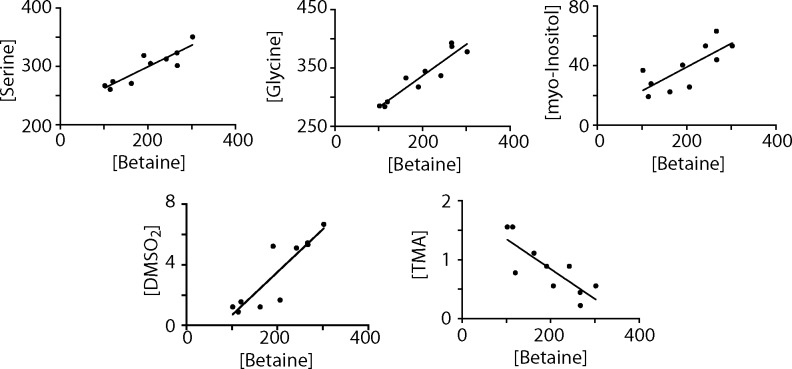
Correlation analyses of metabolites involved in betaine metabolism.

Principal component analysis (PCA) of the 60 quantified serum metabolites revealed a separation by the second principal component (Fig 5A). In particular, glucose (p = 0.06) was lower in the HF + nopal group compared with the HF group, which is consistent with glucose measurement using the enzymatic colorimetric assay. Compounds involved in choline metabolism; serine (p = 0.02), glycine (p = 0.06), and betaine (p = 0.02) were significantly higher in the HF + nopal compared to the HF group (Fig 5B). A significant negative Pearson correlation between serum betaine and hepatic TAG level was observed (Fig 5C) (R^2^ = 0.67, p = 0.007). Positive correlations between betaine and serine (R^2^ = 0.80, p = 0.0005), glycine (R^2^ = 0.88, p<0.0001), myo-inositol (R^2^ = 0.58, p<0.01), dimethylsulfone (R^2^ = 0.79, p<0.0006), and trimethylamine (R^2^ = 0.66, p<0.0045) were observed (Fig 6). These correlations are particularly interesting, as betaine has been used in several human and animal studies as a dietary strategy to ameliorate hepatic steatosis and improve markers of liver injury [29, 30].

Other serum metabolites that changed when rats consuming a HF diet ingested nopal included arginine (p = 0.02), asparagine (p = 0.06), threonine (p = 0.008), creatinine (p = 0.02), dimethylsulfone (p = 0.008), and myo-inositol (p = 0.008), which were all significantly higher, as well as methanol (p = 0.02) and taurine (p = 0.06), which were significantly lower (Fig 5B).

### Effect of nopal ingestion on intestinal inflammation and oxidative stress

**Fig 7:**
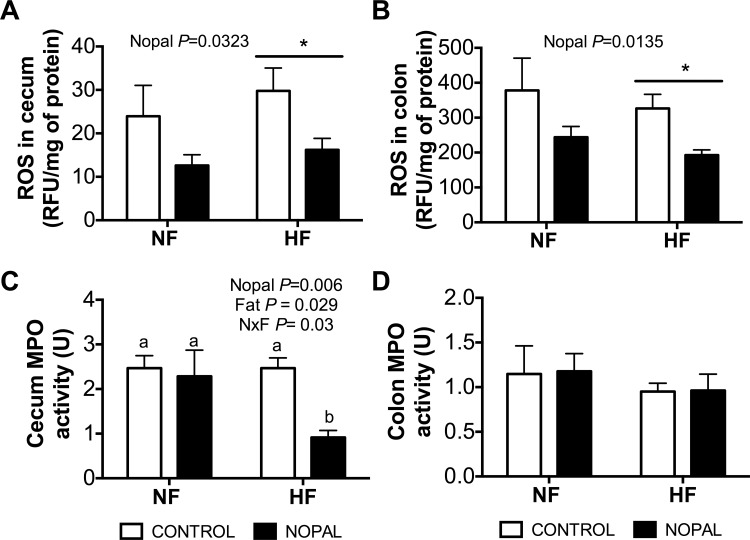
The addition of nopal to the NF and HF diets reduces ROS in the cecum and colon irrespective of the amount of fat. ROS in A) cecum and B) colon. MPO activity of C) cecum and D) colon. Values are presented as mean ± SEM (n = 5–14 per group). Significance was set at p < 0.05.

**Fig 8:**
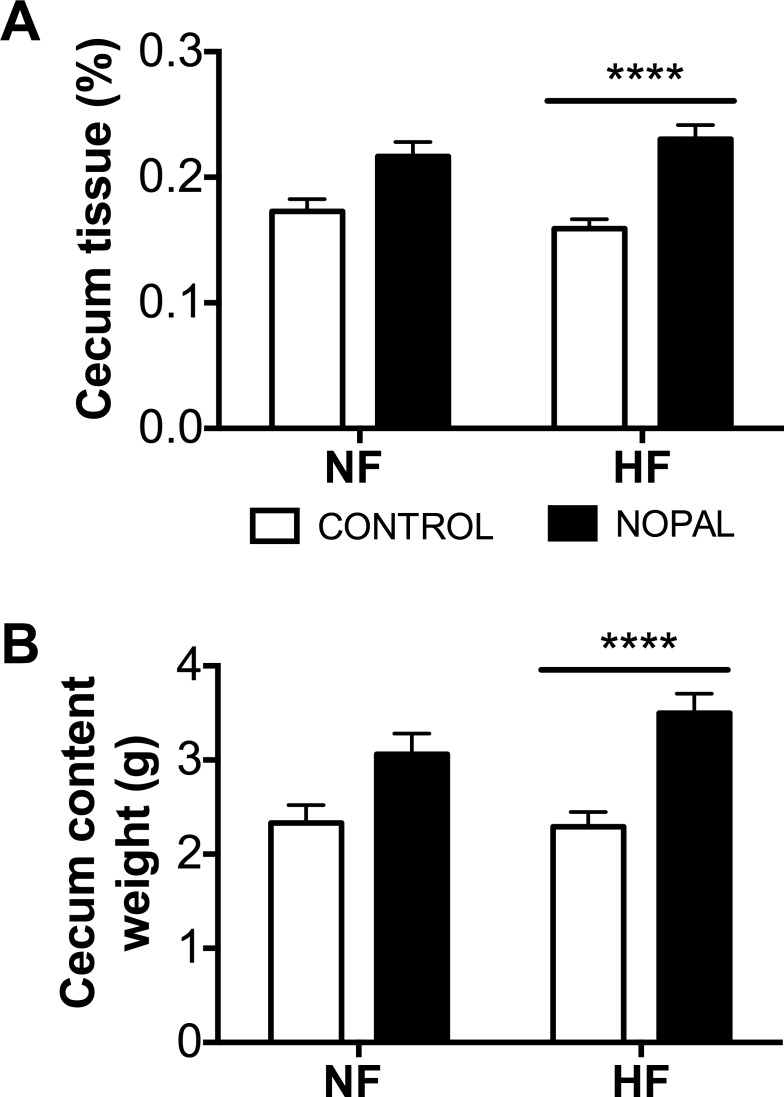
Addition of nopal to the HF diet increases cecum content weight. A) Cecum tissue weight normalized against total body weight. B) Cecum content weight. Data are presented as mean ± SEM (n = 5–14 per group). Values are significantly different **** P < 0.0001.

It has been previously reported that HF diet can lead to oxidative stress and inflammation in the intestine [11, 13]. Thus, relative abundance of reactive oxygen species (ROS), the activity of MPO (as a marker of neutrophil infiltration) and the expression of genes involved in inflammation were measured in the cecal and colonic mucosa. HF diet or nopal consumption had no significant effect on the gene expression of MCP-1 or TNFα (Table 3). However, IL-6 gene expression of colonic but not cecal mucosa was significantly increased by HF diet consumption; this increase was significantly decreased by nopal (Table 3). Rats fed the nopal diets (NF + nopal and HF + nopal) had lower levels of ROS in both the cecal and colonic mucosa compared with rats fed NF and HF diets, respectively (Fig 7A and 7C). However, the difference only achieved significance in HF + nopal fed rats. In addition, cecal MPO activity was significantly lower in HF + nopal compared to HF fed rats (Fig 7B). MPO activity in the colon did not differ between any of the groups (Fig 7D). Interestingly, the weight of cecum tissue and its content mass were both higher in the HF + nopal compared to the HF-fed rats. A similar pattern was also seem when compare NF + nopal to the NF rats but did not reach significant (Fig 8).

**Table 3:** Intestinal mRNA, expression of genes involved in inflammation in Sprague-Dawley rats fed NF, NF + nopal, HF or HF + nopal diets for 10 wk with 12 h of fasting.

	Normal-fat diet		High-fat diet		Two-way anova P value		
	Control	Nopal	Control	Nopal	Nopal (N)	Fat (F)	N x F
**Cecum**							
TNFa relative mRNA	1.39 ± 0.45	1.60 ± 0.52	0.89 ± 0.10	0.87 ± 0.28	NS	NS	NS
IL-6 relative mRNA	1.86 ± 0.77	0.95 ± 0.36	0.51 ± 0.25	0.45 ± 0.05	NS	NS	NS
**Colon**							
TNFa relative mRNA	0.66 ± 0.02	3.34 ± 1.38	1.65 ± 0.69	1.65 ± 0.53	NS	NS	NS
IL-6 relative mRNA	1.11 ± 0.25	0.89 ± 0.43	23.47 ± 3.31	4.91 ± 2.29*	0.02	0.0004	NS
MCP-1 relative mRNA	1.17 ± 0.31	0.61 ± 0.16	1.28 ± 0.17	1.28 ± 0.26	NS	NS	NS

Data are presented as mean ± SEM, n = 5–14

*P < 0.05.

### Nopal ingestion shifted the cecal microbiota

**Fig 9:**
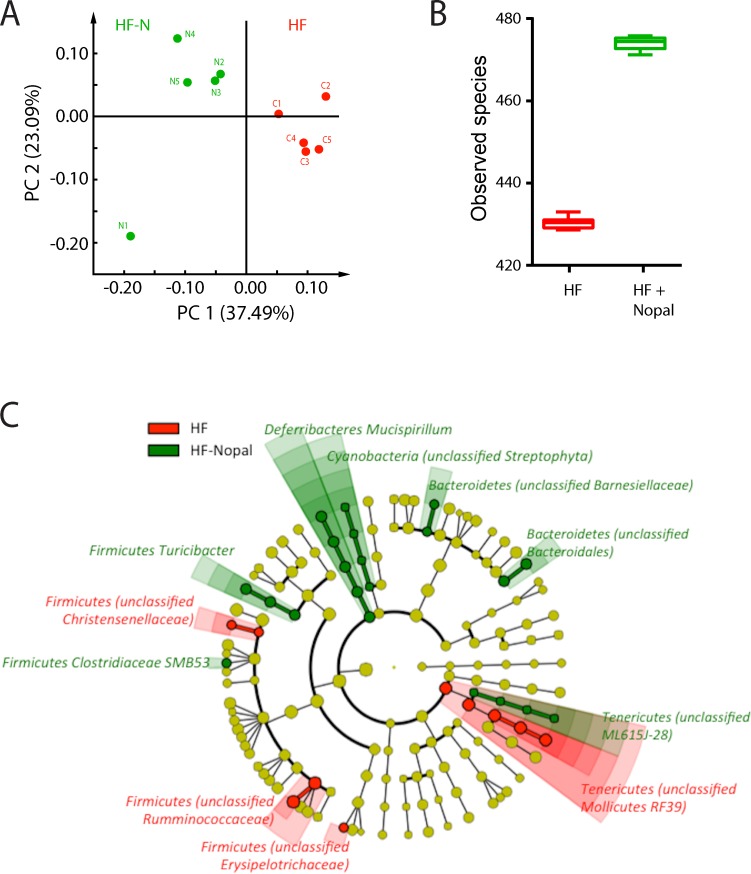
Gut microbiota change with consumption of nopal. A) PCoA of the unweighted UniFrac distances of microbial 16S rRNA sequences, B) Taxonomic richness as measured by number of species observed, C) LEfSe-derived phylogenetic tree depicting nodes within the bacterial taxonomic hierarchy that are significantly enriched in cecal samples from HF (red) and HF + nopal (green) rats. Significant phyla are labeled with the genera in parentheses. LEfSe was used with default parameters. Data are presented as mean ± SEM (n = 5 per group). Values are significantly different * P < 0.05.

To further evaluate the impact of nopal consumption on gut microbiota composition and diversity, 16s microbial rRNA genes from the cecum content of HF and HF + nopal rats (n = 5 per group) were sequenced. Taxonomic levels were determined with the dominant phylum-level representatives of *Firmicutes* (66.7 ± 7.1%) and *Bacteroidetes* (18.8 ± 6.4%) followed by *Tenericutes* (8.7 ± 4.4%). Other phyla such as *Verrucomicrobia*, *Proteobacteria*, *Lentisphaerae*, *Deferribacteres*, *Cyanobacteria*, *Actinobacteria* and *Euryarchaeota* together comprised, on average, less than 6% of the total community composition. To further investigate the effect of nopal consumption on cecal microbial composition, the phylogeny-based Unifrac distance metric analysis was performed to investigate differences in cecal microbial communities between the HF and the HF + nopal rats. The results suggested a significant impact of nopal consumption on the gut microbial profile (Fig 9A). The within-sample α-phylogenetic diversity (observed species) was significantly higher in the nopal rats compared to the HF group (Fig 9B, p = 0.043). Using the linear discriminant analysis (LDA) effect size (LEfSe) method [24], we identified more specific bacterial taxa whose relative abundance varied significantly among cecal samples taken from the HF and HF + nopal groups. Analysis revealed 31 discriminative features (LDA score >3; Fig 9C). Microbiota in the HF group was enriched mainly in taxa within the *Firmicutes* and *Tenericutes* phyla. In the *Firmicutes* phyla, the differentiating families were *Christensenellaceae*, *Ruminococcaceae* and *Erysipelotrichaceae*. Within the *Tenericutes* phyla, the enrichment was in an unclassified family/genera within the *Mollicutes* class. On the other hand, the discriminant features for the HF + nopal group were more diverse and spread amongst different phyla; *Deferibacteres*, *Bacteroidetes*, *Firmicutes*, *Tenericutes* and *Cyanobacteria*. Specifically within the Firmicutes the differentiating genera were *Turicibacter* and SMB53 while within *Defferibacteres* the augmented genera was *Mucillispirum*. For *Bacteroidetes*, *Cyanobacteria* and *Tenericutes*, the differentiating features were mostly unclassified family/genera within *the Bacteroidales*, *Streptohpyta* and ML615J-28 order respectively.

Considering the significant changes in cecum microbial profile, we further evaluated the impact of nopal consumption on intestinal fermentation via ^1^H NMR metabolomics. The aqueous metabolic profiles of rat cecal extracts consist of SCFAs (short chain fatty acids), monosaccharides, amino acids (AAs), phenolic acids, bile acids and other organic acids. Except for butyrate, all SCFAs (acetate, propionate, isobutyrate, isovalerate, and valerate) were significantly higher in the HF + nopal compared to the HF group (Table 4), suggesting an elevation of fermentation activity by nopal ingestion. Succinate as well as several suspected polyphenol break-down products including the quercetin metabolite 3-hydroxyphenylacetate [31], the naringenin metabolite phenylacetate, and the catechin metabolite 3-phenylpropionate [32, 33] were significantly increased in the HF + nopal group (Table 4). Interestingly, higher levels of 2’-deoxyinosine, 2’-deoxyuridine, hypoxanthine, uracil, β-alanine, ribose, and nicotinate, as well as the AAs alanine, glutamate, N-acetylglutamate, and lysine were observed in HF + nopal rats compared to the HF controls. Higher levels of choline, betaine, ornithine, formate, fumarate, glycerophosphocholine, dimethylamine, and bile acids were also observed in the cecal material of the HF + nopal rats. The only metabolite that was higher in the HF mice was xylose, which is likely a breakdown product of cellulose (or hemicellulose) in the HF diet. Interestingly, galacturonic acid was not observed in the cecal content of the HF + nopal fed mice, suggesting it had been completely fermented.

**Table 4:** Cecal metabolites detected by ^1^H NMR spectroscopy in cecal contents of HF and HF + nopal rats. Data are presented as mean ± SEM and expressed as millimoles per gram dry weight. P-values calculated using Mann-Whitney non-parametric test. N = 5 per group.

Compound	HF (mmoles/g_dw_)	HF + nopal (mmoles/g_dw_)	p-Value
**SCFA and polyphenol metabolites:**			
3-Hydroxyphenylacetate	0.059 ± 0.022	5.15 ± 0.38	0.008
3-Phenylpropionate	0.187 ± 0.010	0.427 ± 0.094	0.03
Phenylacetate	0.389 ± 0.130	3.573 ± 0.666	0.008
Succinate	1.20 ± 0.36	2.41 ± 0.20	0.09
Acetate	179.7 ± 22.1	293.1 ± 30.3	0.02
Butyrate	35.79 ± 3.73	39.88 ± 3.20	NS
Propionate	32.69 ± 2.98	57.13 ± 6.12	0.008
Isobutyrate	2.34 ± 0.27	8.05 ± 0.97	0.008
Isovalerate	1.17 ± 0.21	5.29 ± 0.92	0.008
Valerate	4.21 ± 0.30	7.35 ± 0.78	0.008
**DNA metabolism:**			
2’-Deoxyinosine	0.064 ± 0.012	0.181 ± 0.029	0.008
Hypoxanthine	1.53 ± 0.91	2.16 ± 0.14	0.02
2’Deoxyuridine	0.124 ± 0.014	0.281 ± 0.048	0.03
Uracil	2.23 ± 0.17	3.27 ± 0.18	0.008
β-Alanine	0.072 ± 0.008	0.180 ± 0.028	0.008
Ribose	4.54 ± 0.36	7.41 ± 0.37	0.008
			
**Amino acid metabolism:**			
Alanine	7.98 ± 0.38	12.5 ± 2.4	0.03
Lysine	2.03 ± 0.15	3.88 ± 0.87	0.02
Glutamate	12.5 ± 1.3	17.8 ± 1.4	0.03
N-Acetylglutamate	0.177 ± 0.027	0.381 ± 0.035	0.008
**Choline metabolism:**			
Choline	0.108 ± 0.016	0.243 ± 0.066	0.03
Betaine	0.051 ± 0.007	0.152 ± 0.050	0.03
Dimethylamine	0.043 ± 0.008	0.158 ± 0.024	0.008
**Others:**			
Citrulline	2.095 ± 0.109	3.409 ± 0.828	N/S
Ornithine	0.148 ± 0.012	0.402 ± 0.068	0.008
Formate	0.654 ± 0.018	0.790 ± 0.051	0.02
Fumarate	0.149 ± 0.014	0.292 ± 0.047	0.008
Glycero-3-phosphocholine	0.047 ± 0.001	0.080 ± 0.010	0.008
Nicotinate	0.680 ± 0.049	1.401 ± 0.076	0.008
Xylose	4.22 ± 0.23	0.648 ± 0.115	0.008
Bile acids*	12.8 ± 1.4	24.0 ± 3.7	0.03

*Expressed as the mean area under the peaks between 0.60 and 0.75 ppm.

## Discussion

We have previously shown that six weeks of nopal consumption significantly reduced hepatic triglyceride content, improved hepatic insulin signaling and biomarkers of oxidative stress, as well as liver function in a genetically predisposed model of obesity and hepatic steatosis (Zucker *fa/fa* rats) [3]. The present study investigated whether consumption of nopal improves the obese phenotype and metabolic responses induced by a HF diet in rodents. The results show that six weeks of nopal consumption prevented HF diet-induced inflammation in the cecum and colon, and limited the development of adiposity and the increase in adipocyte size associated with long-term ingestion of a HF diet. Nopal consumption increased intestinal fermentation and production of SCFA in the cecum of HF diet fed rats. Concomitantly, nopal significantly increased the cecal microbial diversity, and produced profound changes in bacterial composition and the cecal metabolome. Changes in the serum metabolome were also detected. In particular, analysis of the serum metabolome of HF diet fed rats showed that nopal consumption induced an increase in serum betaine concentration, which correlated negatively with triglyceride content in the liver. Taken together, these data suggest that dietary consumption of nopal can improve gastrointestinal and metabolic consequences of a HF diet.

One of the first health effects documented by nopal consumption was on plasma levels of glucose. Frati *et al*. [1] demonstrated that nopal consumption decreased plasma glucose in hyperglycemic subjects. Since then, nopal has been used in Mexican traditional medicine to aid in the treatment of diabetes. Isolated nopal polysaccharides show antihyperglycemic effects in the absence of significant effects on serum insulin levels in animal models [34, 35]. In the present study, although none of the animals developed hyperglycemia (defined as plasma glucose greater than 200 mg/dL), nopal in the diet significantly lowered fasting glucose levels.

The maintenance of rodents on HF diets for the study of obesity, is characterized by an increase in body weight and adipocyte hypertrophy [36]. In the present study, the HF diet did not result in a significant increase in body weight, but did increase adiposity index and plasma levels of leptin. Addition of nopal to the diet restored adiposity and plasma leptin levels to normal values. Histological evaluation of subcutaneous adipose tissue showed an enlargement of adipocytes in rats ingesting a HF diet, and that addition of nopal to the diet significantly decreased adipocyte size. Analysis of the distribution of subcutaneous adipocyte size further suggests that the decrease of adiposity was due to a reduction in the number of large adipocytes. Furthermore, large adipocytes are characterized by altered insulin sensitivity and higher secretion of leptin [27, 37]. Larger adipocytes have been shown to correlate with a higher degree of inflammation, with more macrophage infiltration and higher overall grade of inflammation in the adipose tissue [27].

**Fig 10:**
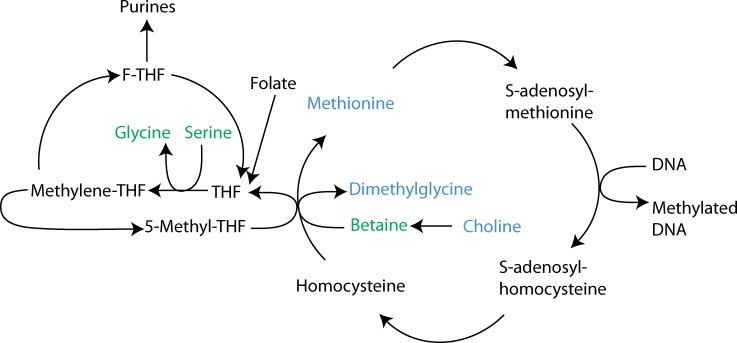
Metabolic pathway of betaine. Metabolites that are higher in HF + nopal rats are indicated in green, those that are lower are in red, and those not significantly different are highlighted in blue. Metabolites that were not measured are in black.

In view of our correlation analysis between serum betaine and hepatic TAG, we postulate that nopal consumption increases serum betaine levels through conversion of absorbed choline to betaine in the liver as part of 1-carbon metabolism (Fig 10). Betaine supplementation has been used in human and animal studies to ameliorate non-alcoholic fatty liver disease and its associated metabolic abnormalities [38]. A recent report showed in humans that serum betaine levels correlated negatively with NAFLD scores [39]. Although negligible amounts of betaine were found in nopal, a significant amount of choline was detected. A positive correlation between betaine, glycine and serine was observed which might suggest that these metabolites are correlated through 1-carbon metabolism (Fig 10). Interestingly, alteration of the 1-carbon pathway could affect methylation of the *PPARα* gene. A positive relationship between hepatic betaine levels and methylation levels of the hepatic *PPARα* promoter have been reported [40]. These results may provide a plausible contributing mechanism to the results observed in our previous study in Zucker (*fa/fa*) rats, as it has been reported that these rats have impaired methionine metabolism combined with lower levels of liver betaine [41].

It has been shown that in rodent models of HF diet-induced obesity, intestinal inflammation precedes weight gain and increased adiposity, suggesting that intestinal inflammation is an early consequence of consumption of a HF diet, and may have a causative role in the onset of obesity [13]. De la Serre *et al*. [8] showed an increase in ileal MPO activity after 8 weeks on a HF diet that was only seen in obese-prone and not obese-resistant rats, suggesting that the obese-phenotype is associated with intestinal inflammation. Furthermore, intestinal inflammation is correlated with metabolic abnormalities associated with the consumption of a HF diet [13]. In the present study, we observed that a HF diet induced a dramatic increase in the expression of IL6 in the colon and that addition of nopal to the diet significantly attenuated this increase. Contrary to previous reports [9], there were no changes in the amount of ROS or MPO activity in cecum and colon between the normal-fat and high fat diet; however, the addition of nopal to either diet induced a decrease in ROS in both the cecum and colon. Additionally, there was a decrease in MPO activity in the cecum in HF + nopal rats compared to HF rats. We also observed increased 2’-deoxyinosine, hypoxanthine, 2’-deoxyuridine, uracil, β-alanine and ribose in the cecal contents of rats fed HF + nopal compared with their HF-fed counterparts. 2’-Deoxyinosine, hypoxanthine and ribose are the products of degradation of the purine deoxyadenosine; and 2’-deoxyuridine, uracil, β-alanine and ribose are the products of degradation of the pyrimidine deoxycytidine. Typically, nucleosides and their digestion products are absorbed in the small intestine through specific nucleoside transporters. That higher levels of nucleosides are observed in the cecum of the rats fed nopal suggest that transport of nucleosides and potentially adenosine deaminase may be down-regulated in the small intestine. It has previously been suggested that inhibition of adenosine deaminase can attenuate mucosal inflammation through reducing mucosal myeloperoxidase activity [42]. Alternatively, changes in cecal microbiota may cause an increase in the metabolism of DNA by the microbes themselves. Distinguishing between these two mechanisms will need to be addressed in a separate study.

Gut fermentation is translated in rodents by a trophic effect on the cecum that seems to be an adaptation of the gut microbiota by changes in number of bacteria, and their production of short chain fatty acids [43]. It has been previously reported in rodents that prebiotic fibers, such as inulin-type fructans, arabinoxylans and other dietary fiber-like components, can counteract the development of increased adiposity, and in parallel, a cecum enlargement is observed [43–45]. In these and other studies, it has been proposed that changes in the profile of the gut microbiota and SCFA production may modulate host adiposity. Here we show that nopal consumption whether on a NF or HF diet results in an enlarged cecum, as reflected by the change in cecum content and cecum tissue weight. Rats on the HF + nopal diet had increased cecal SCFA as assessed through significantly higher production of propionate, acetate, isobutyrate, isovalerate, and valerate. Interestingly, butyrate production was not higher in the rats fed nopal. Besides SCFAs, other metabolic products can affect host physiology. Of special interest was higher levels of nicotinate (niacin) in the HF + nopal. Nicotinate has been shown to have anti-inflammatory properties in the colon [46] and contribute to the maintenance of intestinal homeostasis [47]. Additionally, nicotinate has been widely used to increase HDL-C, and reduce LDL-C [48].

In models of obesity and chronic inflammation, changes in gut microbiota have been observed [49, 50], suggesting that gut inflammation may be a consequence of a shift in the gut microbiota [9]. In the present study, high-throughput sequencing analysis of 16S rRNA revealed a remarkable increase in bacterial diversity in the cecal contents of rats fed the HF + nopal diet compared with rats fed the HF diet. This finding is of special interest as a reduced bacterial diversity has been observed not only in chronic conditions such as inflammatory bowel disease, but also obesity [51, 52]. In addition, weight reduction either by dietary interventions or bariatric surgery has shown to increase this diversity and in parallel improve metabolic parameters [53, 54]. Bacterial composition showed a variety of differences between HF and HF + nopal groups. An important finding was that some of the taxa previously observed in other HF diet and obese models, such as Mollicutes (previously classified in the Firmicutes phyla) [49, 55], RF39 and Erysipelotrichaceae [56] were no longer enriched in the HF + nopal group. Interestingly, the levels of the later were positively correlated with hepatic triglycerides (r = 0.85 p = 0.04 q_value_ = 0.055) and serum leptin levels (r = 0.779, p = 0.008, q_value_ = 0.055), suggesting that these taxa may be correlated with this metabolic phenotype. Low levels of the genus Turicibacter, have been associated with colitis susceptibility [57, 58]. This bacterial genera was among the enriched taxa in the HF + nopal group. Moreover, Turicibacter levels were negatively correlated with cecum MPO activity (r = -0.782, p = 0.008 q_value_ = 0.055).

Choline can also be metabolized to trimethylamine (TMA) by gut microbiota followed by conversion to TMAO by the liver, increasing cardiovascular risk and limiting choline bioavailability [59, 60]. We observed a negative correlation between betaine and serum trimethylamine (Fig 9), as well as between betaine and Erysipelotrichaceae, which is one of the taxa associated with the production of TMA. It is therefore interesting to speculate that nopal consumption alters community structure in the gut such as to reduce the taxa responsible of converting choline to TMA. Additionally, we observed a positive correlation between betaine and dimethylsulfone, suggesting alterations to sulfur metabolism in the GI tract [61].

In conclusion, this study has shown that six week of nopal consumption in a HF diet counteracts HF-induced adiposity and adipocyte hypertrophy. In addition, nopal consumption produced profound changes in intestinal physiology, and increased bacterial diversity and changes in the composition of gut microbiota, which led to increased fermentation in the cecum, to modulation of inflammatory markers in cecum and colon. Furthermore, the addition of nopal increased serum betaine, arising from its choline content, which may partially explain the previously observed effect of nopal in the development of hepatic steatosis. Although further research is needed to fully characterize the changes in adipose tissue and to determine how the gut microbiota induced by nopal consumption contributes to the observed phenotype, this study provides new evidence that the effects of nopal consumption extends beyond the liver and may mediate early changes in the intestinal physiology.
